# High prevalence of ciprofloxacin resistance in *Escherichia coli* isolated from chickens, humans and the environment: An emerging one health issue

**DOI:** 10.1371/journal.pone.0294043

**Published:** 2023-11-20

**Authors:** Tridip Das, Chandan Nath, Pallabi Das, Keya Ghosh, Tahia Ahmed Logno, Pankqj Debnath, Shuvo Dash, Himadri Shankar Devnath, Shubhagata Das, Md Zohorul Islam

**Affiliations:** 1 Poultry Research and Training Centre, Chattogram Veterinary and Animal Sciences University, Chattogram, Bangladesh; 2 Department of Microbiology and Veterinary Public Health, Chattogram Veterinary and Animal Sciences University, Chattogram, Bangladesh; 3 Chattogram Maa-O-Shishu Hospital, Chattogram, Bangladesh; 4 Department of Molecular Life Science, Faculty of Biology, Friedrich Schiller University Jena, Jena, Germany; 5 Department of Pharmacy, University of Science and Technology Chittagong, Chattogram, Bangladesh; 6 250 Bedded General Hospital, Chattogram, Bangladesh; 7 School of Agricultural, Environmental and Veterinary Sciences, Faculty of Sciences and Health, Charles Sturt University, Wagga Wagga, New South Wales, Australia; 8 Department of Veterinary and Animal Sciences, University of Copenhagen, Frederiksberg C, Denmark; Tribhuvan University, NEPAL

## Abstract

The emergence of antimicrobial resistance in commensal bacteria poses a serious public health burden worldwide. Commensals can disseminate the resistance genes to pathogenic bacteria causing life-threatening infections. This cross-sectional study was designed to investigate the antimicrobial resistance pattern and molecular mechanism(s) of ciprofloxacin resistance in commensal *E*. *coli* from three major one health components (humans, animals and the environment) in Bangladesh. Samples were randomly collected from broiler chickens, broiler farm environments and hospitalized human patients from the same geographical area. Isolation and identification of *E*. *coli* were performed following standard bacteriological techniques. Antimicrobial susceptibility testing (AST) was performed by disk diffusion and broth microdilution methods. Mutation at the quinolone-resistance determining region (QRDR) was analyzed by sequencing. Of 450 samples, a total of 287 (63.8%; 95% CI 59.2–68.1%) *E*. *coli* strains was isolated, where 240 (83.6%; 95% CI 78.9–87.5%) strains were phenotypically resistant to ciprofloxacin. The prevalence of ciprofloxacin-resistant *E*. *coli* in broiler chicken, broiler farm environments and hospitalized human patients are 77.6%, 88.8% and 89% respectively. In AST against nine antimicrobials, all the isolates were found to be multidrug-resistant (MDR). The minimum inhibitory concentration (MIC) of ciprofloxacin was ranged from 4 to >128mg/L. Point mutations were detected in several sites of QRDR, specifically at 83 and 87 amino acid positions in *gyrA* gene, and 56, 57, 78, 80 and 84 amino acid positions in *parC* gene. Mutations resulted in amino acid substitutions. Phylogenetic analysis of *gyrA* and *parC* gene sequences showed a close relationship between the strains isolated from different sources. This study demonstrates a high prevalence of ciprofloxacin resistance in commensal *E*. *coli* in humans, animals and environment interface and their genealogically similarity poses an alarming public health consequence.

## Introduction

Antimicrobial resistance (AMR) is an urgent public health concern worldwide. The emergence and dissemination of AMR is an inevitable side effect of the irrational use of antimicrobials in animals and humans. The resistance phenotype disseminates not only into the pathogen but also into the commensal bacteria, constituting a reservoir of resistance genes for potentially pathogenic ones [1, 2]. *E*. *coli* is one of the versatile commensals in warm-blooded animals’ intestinal tract. Commensal *E*. *coli* has shown resistance against numerous antimicrobials due to their widespread use in animals [3, 4] and humans [5, 6]. This organism can transfer resistance traits to other closely related bacterial pathogens via plasmids, making them difficult to kill by common antimicrobials [7].

Quinolones are broad-spectrum antibiotics used in human and veterinary medicine to treat Gram-negative bacterial infections. Ciprofloxacin is one of the quinolone antibiotics commonly used to treat critical bacterial infections in humans. Therefore, WHO listed this antibiotic as one of the most effective and safe medicines needed in a health system [8]. However, inappropriate use of ciprofloxacin results in bacterial resistance development against this valuable antibiotic.

When ciprofloxacin was first introduced, resistance was practically non-existent. However, the widespread use of this antibiotic in both humans and animals as either disease prevention or feed additives results in the emergence of ciprofloxacin-resistant strains of *E*. *coli*. Ciprofloxacin resistance has been reported as high or extremely high in human and food-producing animal isolates from several countries [9]. Although the use of antimicrobials is strictly regulated in developed countries, there is no strict regulation of antimicrobial use in developing countries like Bangladesh. As a result, the emergence of antimicrobial resistance to critically important antibiotics like ciprofloxacin is common in these countries [10, 11].

Although little works have been carried out with *E*. *coli* to detect the ciprofloxacin resistance profile with molecular confirmation, more concise work has yet to be done to evaluate the status of resistance genes in both humans and animals. Therefore, we aimed to detect the resistance pattern and molecular mechanisms of ciprofloxacin resistance in commensal *E*. *coli* from three major one health components (humans, animals and the environment) in Bangladesh.

## Materials and methods

### Study area and population

**Fig 1:**
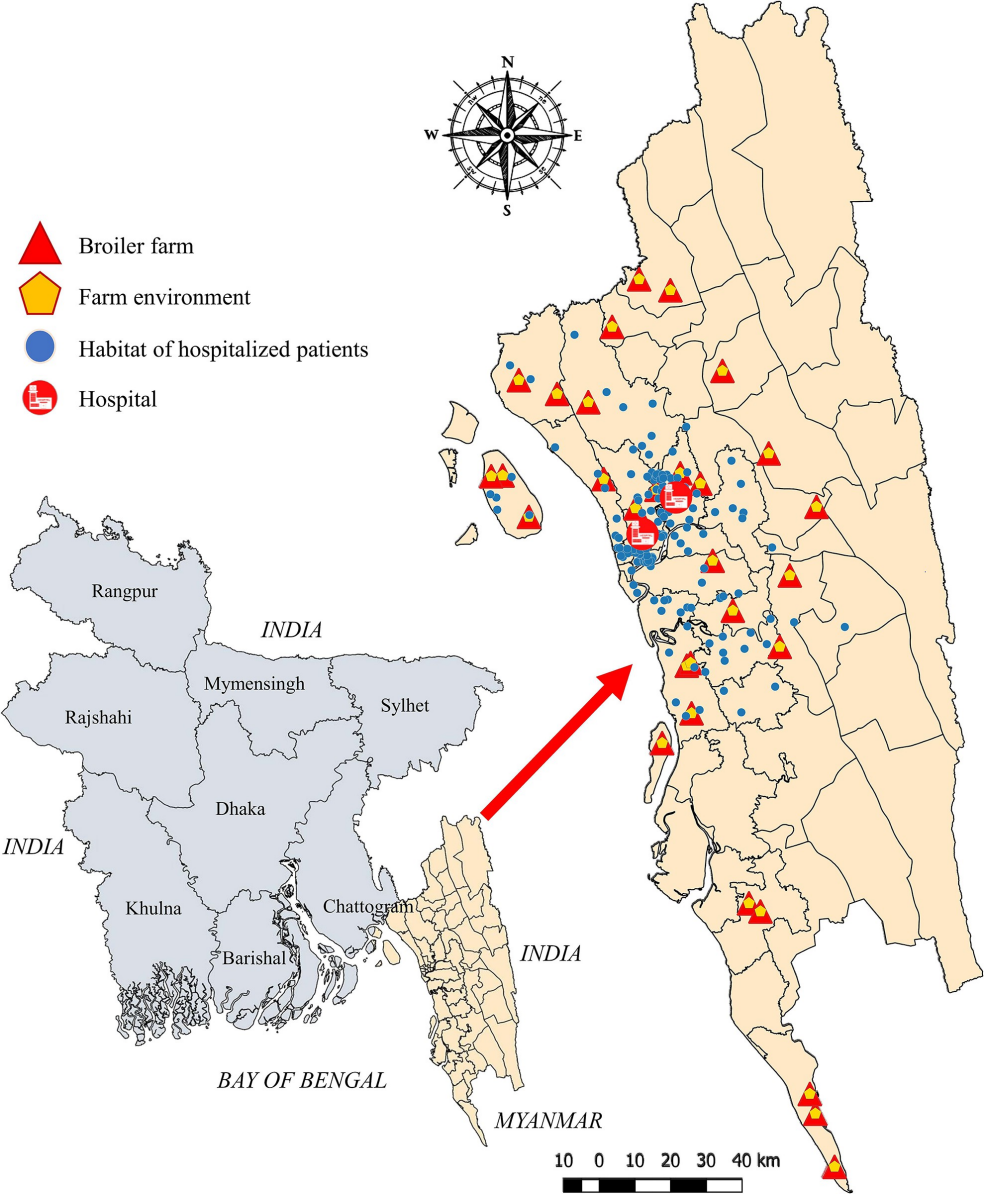
Geographical distribution of sampling sites and population samples. Sample types are marked in the top left legends.

A cross-sectional study was carried out through January to July 2021 in the Chattogram division, located in the southeast region of Bangladesh. Samples from broiler chicken and broiler farm environments were collected from 30 randomly selected broiler farms of eighteen upazilas (administrative sub-district) in Chattogram, Bangladesh. The random selection of broiler farms was obtained from the government livestock office of each upazila. The human samples were collected from two randomly selected human hospitals in the same geographic area (one from a regional area and another from Chattogram metropolitan area). Samples were collected from the hospitalized human patients admitted into both hospitals with gastrointestinal disorders. The geographical distribution of the study area and samples from three different sources are demonstrated in Fig 1. The coordinates of broiler farms and habitat of the hospitalized patients were collected during sampling.

### Sampling

Cloacal swabs from broiler chickens and rectal swabs from hospitalized human patients were collected using sterile cotton swabs. Environmental samples from each broiler farm were collected from feed, water, soil, farm floor litter, and dumping sites. Swab samples were immediately kept in Stuart’s transport medium (Oxoid, Basingstoke, UK) and transferred into the Poultry Research and Training Centre, CVASU, Bangladesh laboratory. Finally, all swab samples were stored at −80°C for further use.

### *E*. *coli* isolation

Isolation and identification of *E*. *coli* were performed following standard bacteriological procedure. Swab samples were placed into 5mL MacConkey broth. Simultaneously, 5g of solid samples (e.g., feed, litter, soil) and 5mL of water samples were taken in separate sterile falcon tubes containing 45 mL MacConkey broth (Oxoid Ltd, Basingstoke, UK) and incubated at 37°C overnight for pre-enrichment. After incubation, one loopful broth was inoculated onto MacConkey agar (Oxoid Ltd, Basingstoke, UK) and incubated at 37°C overnight. Any suspected large pink colony from the plate was subcultured onto Eosin-methylene blue (EMB) agar (Scharlab, Spain) and incubated at 37°C overnight for biochemical confirmation. Colonies with a characteristic greenish metallic sheen from EMB agar were further subcultured onto blood agar (Oxoid Ltd, Basingstoke, UK) with 5% bovine blood and incubated at 37°C for 24 hours. All culture-positive isolates were subjected to *E*. *coli* species confirmation by polymerase chain reaction (PCR) assay targeting a house-keeping gene adenylate kinase (*adk)* using specific primers (AdkF: 5’-ATTCTGCTTGGCGCTCCGGG-3’ and AdkR: 5’- CCGTCAACTTTCGCGTATTT-3’). The thermal condition maintained as initial denaturation at 95°C for 2 minutes and final extension at 72°C for 5 minutes with the 35 cycles of denaturation at 95°C for 1 minute, annealing at 54°C for 1 minute and extension at 72°C for 2 minutes [2].

### Antimicrobial susceptibility testing of *E*. *coli* isolates

Antimicrobial susceptibility testing for all *E*. *coli* isolates was carried out using disk diffusion technique recommended by the Clinical Laboratory and Standards Institute (CLSI) [12, 13]. The ATCC 25922 was used for quality control during the disk diffusion technique. A total of nine antimicrobials from eight different groups were used at the given concentration: colistin sulphate (10 μg), ciprofloxacin (5μg), tetracycline (30μg), ampicillin (10μg), gentamycin (10μg), enrofloxacin (5μg) ceftriaxone (30μg), chloramphenicol (30μg) and sulfamethoxazole/trimethoprim (23.75 + 1.25 μg) (Oxoid, Basingstoke, UK). The results of susceptibility testing were interpreted according to the CLSI guidelines [12, 13].

### Detection of Minimum Inhibitory Concentration (MIC) of ciprofloxacin

All ciprofloxacin-resistant *E*. *coli* isolates were subjected to MIC determination by broth microdilution (BMD) method following ISO standard (20776–2) [14]. Cation-adjusted Mueller Hinton II Broth (Sigma-Aldrich, St Louis, MO, USA) and pure ciprofloxacin powder (Sigma-Aldrich, Saint Louis, MO, USA) were used. A ciprofloxacin resistant *E*. *coli* in-house strain and ATCC 25922 strain were used as the positive and negative control, respectively.

### Gene sequencing and mutation analysis

All phenotypically ciprofloxacin resistant *E*. *coli* isolates were further investigated for the presence of *gyrA* and *parC* genes. The oligonucleotide primers used for the amplification of the genes by PCR are mentioned in Table 1. The thermal cyclic conditions for both genes consisted of an initial denaturation step of 95°C for 3 minutes, followed by 30 cycles of 95°C for 45 sec 56°C for 45 sec and 72°C for 90 sec with a final step of 72°C for 10 minutes. The PCR products were visualized on a gel documentation system (UVP UVsolo touch–Analytik Jena AG) after electrophoresis with 1.5% agarose gel (Thermo Scientific).

**Table 1:** Oligonucleotide primer sequences used to detect the *gyrA* and *parC* genes in the *E*. *coli* isolates.

Gene	Primer name	Primer sequence (5΄- 3΄)	Annealing temperature (°C)	Amplicon size (bp)	References
*gyrA*	gyrA-F	TGGATTATGCGATGTCGGTCAT	56°C	696	This study
	gyrA-R	TTTTGGCGTCAACTTCCACTTC			
*parC*	parC-F	TGCCGTTTATTGGTGATGGTCT	56°C	477	This study
	parC-R	GAGCCACTTCACGCAGGTTAT			

Twenty *E*. *coli* isolates carrying both *gyrA* and *parC* genes were randomly selected for sequencing of both genes. The purified PCR products were Sanger-sequenced with the BigDye terminator v3.1 sequencing kit and a 3730xl automated sequencer (Applied Biosystems, Foster City, CA, USA) using the similar primer sets (Table 1). All sequences were checked for BLASTn and submitted to NCBI for accession number. Geneious prime (version 2022.1.1, Biomatters, New Zealand) software was used for multiple alignments of the sequences from this study with the reference sequence to detect the mutation in QRDR.

### Phylogenetic analysis

Maximum likelihood (ML) based phylogenetic reconstruction was used to determine the genealogical relatedness of both *gyrA* and *parC* amplicons within the study sequences rooting with the *E*. *coli* strain as a reference sequence (NC_007779). For both genes, selected individual sequences were annotated with accession number, strain name, host, country of circulation, collection year and sample ID. A multiple alignment of twenty-one sequences was generated in Geneious with Muscle v3.8.31 alignment algorithm implemented in the Geneious package. For both *gyrA* and *parC* sequences alignments, jModelTest 2.1.3 favoured a general-time-reversible model with gamma distribution rate variation and a proportion of invariable sites (GTR+G+I) for phylogeny. ML trees were reconstructed with 100 bootstrap replicates in PhyML 3.3 implemented in Geneious using the above parameters. The final trees were visualized and edited in FigTree 1.4.

### Statistical analysis

Laboratory data were entered into Microsoft Excel 2016 spreadsheets. The prevalence of all categorical variables and 95% confidence intervals were calculated by the modified Wald method using GraphPad Quickcals (https://www.graphpad.com/quickcalcs/confInterval1/). The heatmap and dendrogram of the antimicrobial susceptibility testing phenotype of *E*. *coli* was generated using the R package ‘gplots’ version 3.0.1 [15].

### Ethics statement

This study was approved [Memo# CVASU/Dir(R&E) EC/2019/39(2/6/6)] by the Ethics Committee of Chattogram Veterinary and Animal Sciences University (CVASU), Bangladesh.

## Results

### Demographic data

A total of 450 samples were collected from the study population. Among them, 150 cloacal swabs from broiler chickens, 150 samples of farm environments and 150 rectal swabs from hospitalized humans. Samples from different environmental matrices are described in Table 2. Although human samples were collected from two hospitals, the hospitalized patients were inhabited in 19 upazilas (Fig 1). Around 43% (65/150) of broiler chicken samples were from the smallholding farms (less than 2500 farm size) as well as the age group of 14 to 24 days (S1 Table). Surprisingly all the farms sampled had a record of antibiotic use, especially ciprofloxacin. The majority of the farms sampled use sawdust as litter materials (90%) and reuse them (S2 Table). About 53.3% of tested farms dump litter materials in nearby open places to the farms. Dumped litters were primarily connected with either canal (23.3%) or crop field (23.3%). Regarding hospitalized human samples, the highest proportion was from the age group 18 to 55 years. Around 59.3% of the sampled patients were male, and almost half of all patients were suffering from diarrhoea (S3 Table). Surprisingly, 71.3% of the human patients sampled had a history of using ciprofloxacin.

**Table 2:** Distribution of *E*. *coli* and ciprofloxacin resistant *E*. *coli* isolated from poultry, poultry farm environment and hospitalized human samples.

Sample source	N	*E*. *coli* (%; 95% CI)	Ciprofloxacin resistant *E*. *coli* (%; 95% CI)
Poultry (broiler chicken)	150	134 (89.3; 83.3–93.4)	104 (77.6; 69.8–83.9)
Poultry farm environment	150	80 (53.3; 45.4–61.1)	71 (88.8; 79.8–94.1)
Litter	30	12 (40; 24.6–57.7)	12 (100; 71.8–100)
Soil	30	14 (46.7; 30.2–63.9)	9 (64.3; 38.6–83.8)
Feed	30	15 (50; 33.2–66.9)	12 (80; 54.1–93.7)
Water	30	23 (76.7; 58.8–88.5)	22 (95.6; 77.3–99.9)
Dumped litter	30	16 (53.3; 36.1–69.7	16 (100; 77.3–100)
Hospitalized human	150	73 (48.7; 40.8–56.6)	65 (89; 79.6–94.6)
**Total**	**450**	**287 (63.8; 59.2–68.1)**	**240 (83.6; 78.9–87.5)**

Here, N = number of samples, % = percentages and CI = confidence interval.

### Prevalence of *E*. *coli*

A total of 287 (63.8%; 95% confidence interval (CI) 59.2–68.1%) samples were tested positive for the presence of *E*. *coli* (Table 2). The highest prevalence of *E*. *coli* was found in broiler chickens (89.3%, 95% CI 83.3–93.4%) followed by farm environments and hospitalized humans. as observed for broilers, farm environment (53.3%, 95% CI 45.4–61.1%) and hospitalized humans (48.7%, 95% CI 40.8–56.6%).

The age group of more than 28 days in broiler chicken has the highest prevalence of *E*. *coli* (98.3%) and ciprofloxacin-resistant *E*. *coli* (S1 Table). Drinking water collected from the water pan has the highest prevalence of *E*. *coli* (76.7%) and ciprofloxacin *E*. *coli* (73.3%) among other matrices (S2 Table). The prevalence of *E*. *coli* (54.1%) and ciprofloxacin-resistant *E*. *coli* (47.4%) were high in the farm environments of those farms reused litter materials. In hospitalized humans, the highest prevalence of *E*. *coli* (71.5%) and ciprofloxacin *E*. *coli* (67.3%) were isolated from the age group of more than 55 years. Male patients had the highest prevalence of *E*. *coli* (53.9%) and ciprofloxacin-resistant *E*. *coli* (46.1%). Simultaneously, the prevalence of *E*. *coli* (50.6%) and ciprofloxacin-resistant *E*. *coli* (44.3%) were high in non-diarrhoeic patients. A total of 48.6% samples from hospitalized humans those used ciprofloxacin for medication, were positive for ciprofloxacin resistant *E*. *coli* (S3 Table).

### Antimicrobial resistance profile of *E*. *coli*

**Fig 2:**
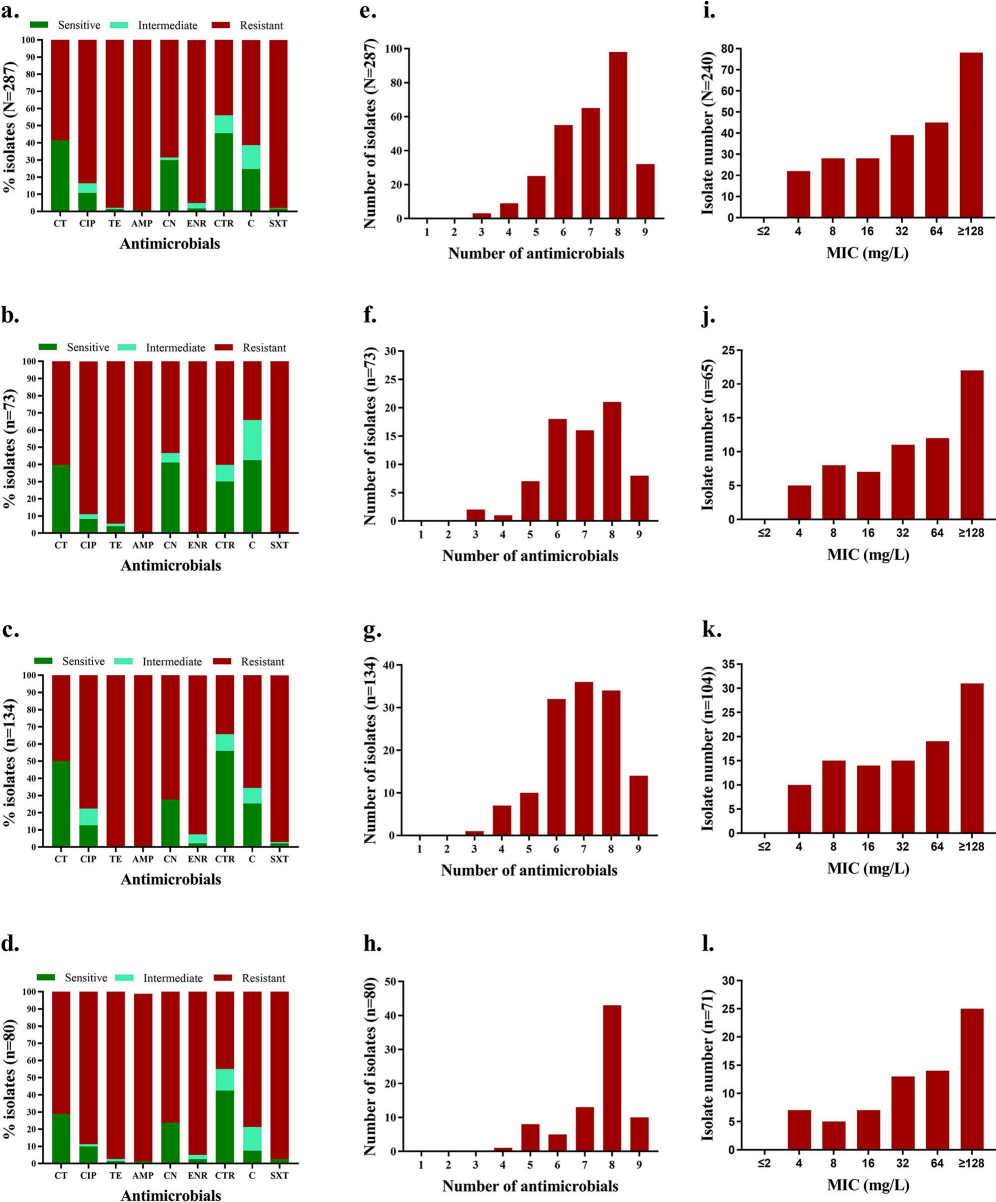
Antibiogram profile of *E*. *coli* isolates isolated from different sources. (a) Overall susceptibility pattern of *E*. *coli* by disc diffusion method; (b) Susceptibility pattern in *E*. *coli* from human patients; (c) Susceptibility pattern in *E*. *coli* from broiler chickens; (d) Susceptibility pattern in *E*. *coli* from farm environment; (e) Overall multidrug-resistant pattern; (f) Multidrug-resistant pattern of *E*. *coli* from human patients; (g) Multidrug-resistant pattern of *E*. *coli* from broiler chickens; (h) Multidrug-resistant pattern of *E*. *coli* from farm environments; (i) Overall MIC of *E*. *coli;* (j) MIC of *E*. *coli* from human patients; (k) MIC of *E*. *coli* from broiler chickens; (l) MIC of *E*. *coli* from farm environments. Here, CT = Colistin Sulphate, CIP = Ciprofloxacin, TE = Tetracycline, AMP = Ampicilin, CN = Gentamycin, ENR = Enrofloxacin, CTR = Ceftriaxone, C = Chloramphenicol, SXT = Sulfamethoxazole/Trimethoprim.

Overall antimicrobial susceptibility patterns of the *E*. *coli* strains to nine antimicrobials are presented in Fig 2. The highest resistance rate of *E*. *coli* was to ampicillin (99.3%), followed by sulfomethoxazole (97.9%), and tetracycline (97.9%) (Fig 2A). *E*. *coli* strains isolated from the human patients show the highest resistance against ampicillin (100%), enrofloxacin (100%) and sulfamethoxazole (100%) (Fig 2B). In contrast, strains isolated from the broiler chickens and the farm environment show the highest resistance against tetracycline (100% and 98.8%, respectively) (Fig 2C and 2D). On the other hand, the overall highest sensitivity was found to ceftriaxone (45.6%) followed by colistin sulphate (41.5%) and gentamycin (30%).

Substantially, 83.6% (95% CI 78.9–87.5%) isolates were resistant to ciprofloxacin. The highest proportion of ciprofloxacin resistant *E*. *coli* isolates were detected in hospitalized humans (89%) (Fig 2B), where the proportion of ciprofloxacin resistant *E*. *coli* isolates in broiler and broiler farm environments were 77.6% and 88.8%, respectively (Fig 2C and 2D).

All the isolates were found to be multidrug-resistant (MDR) as they showed resistance to more than two antimicrobials tested (Fig 2E). Most isolates were resistant to more than six antimicrobials tested (Fig 2E–2H). A detailed outline of antimicrobial resistance phenotypes in *E*. *coli* strains from different sources is illustrated in heatmaps (S1 Fig). The dendrogram on the left side revealed the clustering of the isolates according to their antimicrobial resistance phenotypes.

### MIC of ciprofloxacin

The MIC of all ciprofloxacin-resistant isolates (n = 240) was tested. Overall, 32.5% of the tested isolates show extremely high MIC values (≥128mg/L) against ciprofloxacin (Fig 2I). Alarmingly, 33.8% of ciprofloxacin-resistant *E*. *coli* strains from human patients showed very high MIC (≥128mg/L) (Fig 2J). The *E*. *coli* strains from broiler chickens show moderately lower MIC values compared with the environmental samples (Fig 2K and 2I).

### Gene sequencing and mutations at QRDR

A total of 240 phenotypically ciprofloxacin-resistant isolates were selected for PCR assays to detect *gyrA* and *parC* genes. All the isolates were positive for *gyrA* and *parC* genes. As mutation(s) in the *gyrA* and *parC* genes, specifically in QRDR results in the development of resistance to the ciprofloxacin, we detected point mutation(s) in these two genes. Twenty isolates were selected randomly, covering all types of sample sources for sequencing the *gyrA* and *parC* genes. The gene sequences were submitted to the NCBI nucleotide sequence database, which are available under the accession number ON463777-96 for the *gyrA* gene and ON479036-65 for *parC* gene.

We detected point mutations at several sites of QRDR, specifically 83 and 87 amino acid positions in *gyrA* gene (Table 3). At 83 position *gyrA* genes of all sequences, serine (TCG) was substituted by leucine (TTG) due to a single point mutation in the amino acid codon. However, at 87 position aspartic acid (GAC) was replaced by either histidine (CAC) or asparagine (AAC) or glycine (GGC) or tyrosine (TAC).

**Table 3:** Mutations patterns in ciprofloxacin resistant *E*. *coli* isolates with AST zone and MIC value.

Isolate name	Inhibition zone (mm) of ciprofloxacin	MIC mg/L of ciprofloxacin	Amino acid (codon) at indicated position of Quinolone-Resistance Determining Region								
			*gyrA* accession	*gyrA* 83	*gyrA* 87	*parC* accession	*parC* 56	*parC* 57	*parC* 78	*parC* 80	*parC* 84
*E*. *coli* ATCC strain	34	0.02	NC_007779	Ser(TCG)	Asp(GAC)	NC_007779	Ala(GCC)	Ser(AGC)	Gly(GGC)	Ser(AGC)	Glu(GAA)
EC/Chicken-CS009	6	>128	ON463777	Leu(TTG)	His(CAC)	ON479036	–	–	–	Ile(ATC)	Gly(GGA)
EC/Chicken-CS031	13	32	ON463778	Leu(TTG)	Asn(AAC)	ON479037	–	Thr(ACC)	Cys(TGC)	Ile(ATC)	–
EC/Chicken-CS045	6	>128	ON463779	Leu(TTG)	His(CAC)	ON479038	Thr(ACC)	–	Cys(TGC)	Ile(ATT)	–
EC/Chicken-CS078	6	>128	ON463780	Leu(TTG)	Gly(GGC)	ON479039	–	–	–	Arg(AGA)	–
EC/Chicken-CS093	6	>128	ON463781	Leu(TTG)	His(CAC)	ON479040	–	–	–	Ile(ATT)	Gly(GGA)
EC/Chicken-CS108	6	>128	ON463782	Leu(TTG)	Asn(AAC)	ON479041	–	–	Cys(TGC)	Ile(ATC)	–
EC/Chicken-CS119	6	64	ON463783	Leu(TTG)	His(CAC)	ON479042	–	Thr(ACC)	–	Ile(ATC)	–
EC/Chicken-CS133	6	>128	ON463784	Leu(TTG)	His(CAC)	ON479043	–	–	–	Thr(ACC)	Ala(GCA)
EC/Feed-CS140	13	32	ON463785	Leu(TTG)	His(CAC)	ON479044	Gly(GGC)	–	Cys(TGC)	Arg(AGA)	–
EC/Soil-CS149	9	64	ON463786	Leu(TTG)	Tyr(TAC)	ON479045	–	–	–	Arg(AGA)	–
EC/Water-CS158	11	64	ON463787	Leu(TTG)	His(CAC)	ON479046	–	–	–	Ile(ATT)	Gly(GGA)
EC/Water-CS159	6	>128	ON463788	Leu(TTG)	His(CAC)	ON479047	–	–	–	Ile(ATC)	–
EC/Feed-CS177	6	>128	ON463789	Leu(TTG)	Asn(AAC)	ON479048	–	Ile(ATC)	–	Ile(ATT)	–
EC/Litter-CS184	14	16	ON463790	Leu(TTG)	Gly(GGC)	ON479049	–	–	–	Arg(AGA)	–
EC/Litter-CS192	6	16	ON463791	Leu(TTG)	His(CAC)	ON479050	–	–	–	Ile(ATC)	Lys(AAA)
EC/Human-CS199	6	>128	ON463792	Leu(TTG)	Tyr(TAC)	ON479051	–	–	Cys(TGC)	Ile(ATC)	–
EC/Human-CS207	6	64	ON463793	Leu(TTG)	Gly(GGC)	ON479052	–	–	–	Ile(ATC)	Gly(GGA)
EC/Human-CS208	6	>128	ON463794	Leu(TTG)	His(CAC)	ON479053	–	Gly(GGC)	–	Ile(ATT)	–
EC/Human-CS217	14	32	ON463795	Leu(TTG)	Asn(AAC)	ON479054	–	Ile(ATC)	–	Arg(AGA)	–
EC/Human-CS226	11	16	ON463796	Leu(TTG)	His(CAC)	ON479055	–	–	–	Ile(ATC)	Ala(GCA)

Here, AST = Antimicrobial susceptibility testing, MIC = Minimum inhibitory concentration, Ser = Serine, Asp = Aspartic acid, Leu = Leucine, His = Histidine, Gly = Glycine, Asn = Asparagine, Thr = Threonine, Cys = Cystine, Ile = Isoleuocine, Glu Glutamate, Arg = Argenine, Ala = Alanine

In QRDR of *parC* genes, mutations were detected in 56, 57, 78, 80 and 84 amino acid positions (Table 3). Single- or double-point mutation was detected at position 80 in all the sequences of the study. These mutations result in the substitution of serine (AGC) into either isoleucine (ATC/ATT) or arginine (AGA), or threonine (ACC). Simultaneously, two of the sequences processed mutations at 56 amino acid positions resulting in changing of alanine (GCC) into either threonine (ACC) or glycine (GGC). However, at 57 position, serine (AGC) was replaced by either threonine (ACC) or isoleucine (ATC) or glycine (GGC). At the 78 position, glycine (GGC) was swapped into cysteine (TGC) in five sequences. Glutamate (GAA) at the 84 position was replaced by either glycine (GGA) or alanine (GCA), or lysine (AAA) in some of the *parC* sequences.

A Common similarity between isolates from three types of sources was the substitution of serine (TCG) by leucine (TTG) with a single point mutation in the 83 amino acid codon of *gyrA* gene. On the other hand, those isolates had the mutation in the 78 amino acid codon of *parC* gene due to a single point mutation, namely glycine (GGC) was swapped into cysteine (TGC).

### Phylogeny

**Fig 3:**
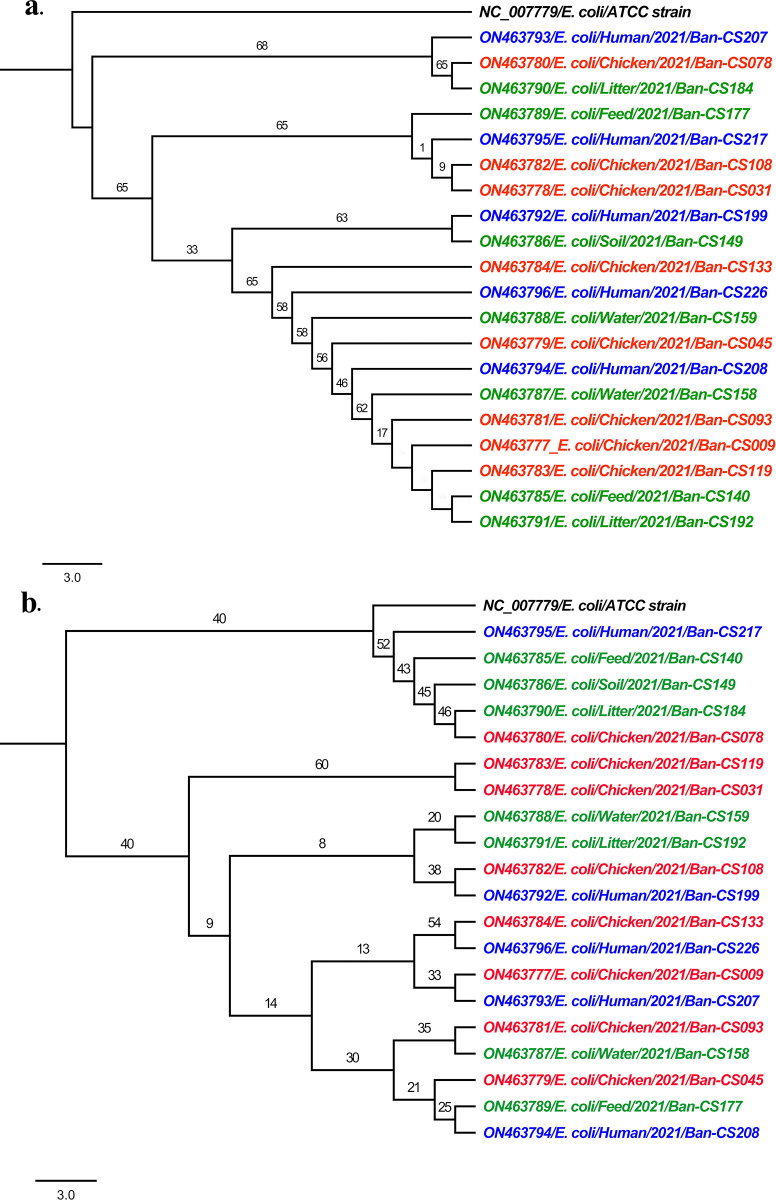
Phylogenetic tree of *gyrA* and *parC* sequences. (a) Maximum likelihood (ML) phylogenetic tree of *gyrA* gene of study sequences rooted with ATCC reference sequence. (a) Maximum likelihood (ML) phylogenetic tree of *gyrA* gene of study sequences rooted with *E*. *coli* reference strain (NC_007779). Here, sequences from poultry (broiler chicken), poultry farm environments, and hospitalized human patients’ origin are highlighted in red, green, and blue, respectively.

Maximum likelihood (ML) phylogenetic tree of twenty *gyrA* gene sequences revealed that all the sequences were grouped into three monophyletic clades (Fig 3A). Each of the clades has an admixture of samples of poultry, farm environment and hospitalized human patient origin indicating their phylogenetic similarities. Conversely, the ML phylogenetic tree of twenty *parC* gene sequences clustered into four monophyletic clades (Fig 3B), where three clades formed with the samples from all three sources and another clade formed with exclusively broiler chicken origin.

## Discussion

AMR is an alarming public health burden worldwide, and it is concerning when commensal bacteria develop resistance to vital antimicrobial agents. Our study generated evidence of a high prevalence of AMR in commensal *E*. *coli* isolated from hospitalized humans and animal sources. This study primarily focuses on the emergence of ciprofloxacin resistance in commensal *E*. *coli* followed by characterization of the mechanism of ciprofloxacin resistance phenomenon by mutation analysis in QRDR.

A high prevalence of *E*. *coli* from broiler chicken (89.3%) and farm environment (53.3%) was recorded, which can be influenced by factors such as age, strains and husbandry system of broiler chicken. For instance, the prevalence of *E*. *coli* is higher in free-range chickens than caged chickens [16]. However, similar to this study, a high prevalence of *E*. *coli* was recorded in chickens in Bangladesh [2–4, 17]. We observed a moderately high prevalence of *E*. *coli* (48.5%) in human diarrhoeal patients compared with previous studies in other countries, including India [18], Myanmar [19], Egypt [20], Iran [21], Mexico [21, 22]. This prevalence can be varied with geographical places due to multiple factors like age, disease condition, the onset of illness, etc.

We detected a high prevalence of ciprofloxacin-resistant *E*. *coli* in broiler chickens as well as in broiler farm environments and hospitalized human patients in Bangladesh. Previous studies in Bangladesh, India, Nepal, Pakistan and Myanmar have shown a comparatively lower prevalence of ciprofloxacin-resistant *E*. *coli* in chickens and hospitalized human patients [4, 19, 23–25]. However, several factors might regulate the prevalence among the geographical locations. The prevalence of ciprofloxacin resistance in commensal *E*. *coli* is alarming because these commensal bacteria can transfer their resistant traits among other enteric pathogens, which might have remarkable public health consequences. Ciprofloxacin is a crucial antimicrobial used to treat enteric infections in humans [8]. The high level of resistance developed in bacteria against this antibiotic indicates the consequence of over and indiscriminate use of this drug not only in the poultry industry of Bangladesh but also in human sectors [4, 26].

Microbes that are resistant to at least three groups or classes of antimicrobials are considered MDR [3, 27]. Alarmingly, all of the *E*. *coli* isolates in this study were found to be MDR. The widespread use of antimicrobials in the poultry and human sectors may be to blame for the high prevalence of MDR in the study area [28]. Previous studies have shown a wide range of MDR *E*. *coli* prevalence from different sources, including 85.7% in humans [29], 62.9% in drinking water [30] and 100% in poultry [26]. The prevalence of MDR *E*. *coli* varied in different age groups. For instance, young children aged between 3–24 years had a moderately low prevalence of *E*. *coli* than adult humans [23]. The common antimicrobials like ciprofloxacin, ampicillin, and tetracycline, used in both humans and broiler chickens found to be resistant against *E*. *coli*. The pattern of MDR *E*. *coli* isolated from humans and broiler chickens was remarkably similar, indicating possible transmission between the sources. Once these MDR isolates can produce any pathogenicity to the host, it will be a horrible situation to tackle the infection using the antimicrobials mentioned above.

Surprisingly, a significant proportion of all phenotypically ciprofloxacin-resistant *E*. *coli* had higher MIC values. According to the CLSI, the breakpoint of ciprofloxacin is 1mg/L. In contrast, most of the isolates in this study, had MIC values of more than 16mg/L, indicating a higher spectrum of resistance to ciprofloxacin by *E*. *coli*. A Similar pattern of high MIC values for ciprofloxacin-resistant *E*. *coli* from samples of diverse origin has been reported in several geographical areas [31–33], which indicates a widespread distribution of ciprofloxacin-resistant *E*. *coli*. The possible factors of such increasing frequency of MIC might be a horizontal and vertical transmission of the genotype within and among the hosts. Another significant predisposing factor, like irrational use of ciprofloxacin might trigger the phenomenon of high MIC value.

Mutations in specific domains of *gyrA*, *parC*, and *parE* cause single amino acid changes in either gyrase or topoisomerase IV that contribute to quinolone resistance. Multiple mutations in the QRDR of topoisomerase enzymes are usually associated with a high-level of fluoroquinolone resistance in *E*. *coli* strains [34]. We detected multiple mutations at QRDR in our study’s *gyrA* and *parC* genes sequences, which resulted in several amino acid substitutions in both genes. Most amino acid substitutions occurred due to single-point mutation at QRDR. This molecular confirmation supports the high level of phenotypic resistance found in this study. Regarding mutations in *gyrA* gene and *parC* gene, broiler chicken and human isolates had highly similar mutation patterns, indicating a commonality of the isolates. Although *gyrA* and *parC* genes of randomly selected isolates were sequenced, to understand the bigger picture of ciprofloxacin resistance, sequencing followed by mutation determination of all isolates might give a detailed mechanistic overview. Phylogenetic analysis also exhibited the genetic similarity of the isolates where they were clustered into similar monophyletic clades. These might indicate a link of transmission of resistant bacteria between humans and animal sources. Horizontal transmission of these resistance genes might be one of the considered factors for such resistance dissemination in one health interphases. Therefore, large scale-systematic study considering all one-health components is warranted to identify the detailed transmission route of antimicrobial resistance.

## Conclusions

We isolated commensal *E*. *coli* from broiler chickens, broiler farm environments, and hospitalized human samples at a high frequency but with variable levels of prevalence. Most isolates obtained from the sources of investigations were found to be MDR. Surprisingly, more than 83% of ciprofloxacin-resistant commensal *E*. *coli* are circulating in one health interphase in Bangladesh carrying acquired mutations in *gyrA*, and *parC* genes. Therefore, it is important to develop awareness against the extensive use of antimicrobials in humans and animals to reduce the spread of MDR bacteria.

## Supporting information

S1 Table Demographic data and prevalence of *E*. *coli* and ciprofloxacin resistant *E*. *coli* in broiler chicken. (DOCX)

S2 Table Demographic data and prevalence of *E*. *coli* and ciprofloxacin resistant *E*. *coli* in broiler farm environment. (DOCX)

S3 Table Demographic data and prevalence of *E*. *coli* and ciprofloxacin resistant *E*. *coli* in hospitalized humans. (DOCX)

S1 Fig Hierarchical clustering dendrogram representing antibiogram of *E*. *coli* isolates isolated from diverse sources. (a) cloacal swab poultry, (b) samples farm environment, (c) human patients [CT = Colistin Sulphate, CIP = Ciprofloxacin, TE = Tetracycline, AMP = Ampicilin, ENR = Enrofloxacin, CRT = Ceftriaxone, CN = Gentamycin, SXT = Sulfamethoxazole & Trimethoprim and C = Chloramphenicol]. (Here letters in the right side of the map represents the sample ID, red color represents resistance profile of the mentioned antimicrobials (lower axis of the map) and green color represents the sensitivity profile. All the intermediately susceptible isolates were considered as susceptible. The dendrogram in the left side clustered the isolates based on their phenotypic antibiogram pattern).
(TIF)

S1 File (DOCX)

S2 File (PDF)
