# Supplementary material for: High prevalence of ciprofloxacin resistance in Escherichia coli isolated from chickens, humans and the environment: An emerging one health issue
Source: PLoS One. 2023 Nov 20;18(11):e0294043. doi: 10.1371/journal.pone.0294043 (PMC10659180; doi:10.1371/journal.pone.0294043)
Supplement: S1 Table — (DOCX) [file pone.0294043.s001.docx]

**S1 Table. Demographic data and prevalence of *E. coli* and ciprofloxacin resistant *E. coli* in broiler chicken**.

| **Explanatory variable** | **Co variable** | ***E. coli*** | | | **Ciprofloxacin resistant *E. coli*** | | |
| --- | --- | --- | --- | --- | --- | --- | --- |
|  |  | **N** | **Prevalence (%; 95% CI)** | ***p*- value** | **N** | **Prevalence (%; 95% CI)** | ***p-* value** |
| Farm size | Less than 2500 | 65 | 56 (86.2; 75.5-92.8) | 0.097 | 56 | 42(75; 62.2-84.6) | 0.826 |
|  | 2500 to 5000 | 55 | 53 (96.4; 87-99.7) |  | 53 | 42(79.2; 66.4-88.2) |  |
|  | More than 5000 | 30 | 25 (83.3; 66-93.1) |  | 25 | 20(80; 60.4-91.6) |  |
| Age | Less than 14 days | 25 | 22(88; 69.2-96.7) | 0.010 | 22 | 17(77.3; 56.1-90.3) | 0.996 |
|  | 14 to 24 days | 65 | 53(81.5; 70.3-89.3) |  | 53 | 41(77.4; 64.3-86.7) |  |
|  | More than 24 days | 60 | 59(98.3; 90.3-100) |  | 59 | 46(77.97; 65.7-86.8) |  |
| Strain | Cobb-500 | 100 | 90(90; 82.4-94.6) | 0.172 | 90 | 71(78.9; 69.3-86.3) | 0.938 |
|  | Lohmann | 15 | 14(93.3; 68.2-100) |  | 14 | 11(78.6; 51.7-93.6) |  |
|  | Ross | 15 | 11(73.3; 47.6-89.5) |  | 11 | 8(72.7; 42.9-90.8) |  |
|  | Habbard classic | 20 | 19(95; 74.6-100) |  | 19 | 14(73.7; 50.9-88.5) |  |
| Water source | Tube well | 75 | 68(90.7; 81.7-95.7) | 0.597 | 68 | 53(77.9; 66.6-86.3) | 0.926 |
|  | Deep tube well | 75 | 66(88; 78.5-93.8) |  | 66 | 51(77.3; 65.7-85.8) |  |
| Litter materials | Saw dust | 135 | 123(91.1; 85-95) | 0.172 | 123 | 94(76.4; 68.1-83.1) | 0.269 |
|  | Rice husk | 15 | 11(73.3; 47.6-89.5) |  | 11 | 10(90.9; 60.1-100) |  |
| Floor type | Muddy | 65 | 58(89.2; 79.1-95) | 0.633 | 58 | 43(74.1; 61.5-83.7) | 0.554 |
|  | Brick | 20 | 19(95; 74.6-100) |  | 19 | 15(78.9; 56.1-92.05) |  |
|  | Concrete | 60 | 52(86.7; 75.6-93.3) |  | 52 | 43(82.7; 70-90.8) |  |
|  | Bamboo | 5 | 5(100; 51.1-100) |  | 5 | 3(60; 22.9-88.4) |  |
| Litter reuse | Yes | 135 | 120(88.9; 82.4-93.2) | 0.597 | 120 | 94(78.3; 70.1-84.8) | 0.558 |
|  | No | 15 | 14(93.3; 68.2-100) |  | 14 | 10(71.4; 45-88.7) |  |
| Ciprofloxacin use | No | 0 | - | - | 0 | - | - |
|  | Yes | 150 | 134(89.3; 83.3-93.4) |  | 135 | 104(77.6; 69.2-83.4) |  |
| Growth promoter use | No | 35 | 30(85.7; 70.1-94.2) | 0.428 | 30 | 23(76.7; 58.8-88.5) | 0.888 |
|  | Yes | 115 | 104(90.4; 83.5-94.7) |  | 104 | 81(77.9; 68.9-84.8) |  |
