# Supplementary material for: High prevalence of ciprofloxacin resistance in Escherichia coli isolated from chickens, humans and the environment: An emerging one health issue
Source: PLoS One. 2023 Nov 20;18(11):e0294043. doi: 10.1371/journal.pone.0294043 (PMC10659180; doi:10.1371/journal.pone.0294043)
Supplement: S2 Table — (DOCX) [file pone.0294043.s002.docx]

**S2 Table. Demographic data and prevalence of *E. coli* and ciprofloxacin resistant *E. coli* in broiler farm environment**

| **Explanatory variable** | **Co variable** | ***E. coli*** | | | **Ciprofloxacin resistant *E. coli*** | | |
| --- | --- | --- | --- | --- | --- | --- | --- |
|  |  | **N** | **Prevalence (%; 95% CI)** | ***p*-value** | **N** | **Prevalence (%) (95% CI)** | ***p*-value** |
| Footbath | Yes | 65 | 37(56.9; 44.8-68.2) | 0.441 | 37 | 34(91.9; 78-97.9) | 0.409 |
|  | No | 85 | 43(50.6; 40.2-61) |  | 43 | 37(86.05; 72.3-93.8) |  |
| Litter materials | Saw dust | 135 | 71(52.6; 44.2-60.8) | 0.585 | 71 | 62(87.3; 77.4-93.4) | 0.257 |
|  | Rice husk | 15 | 9(60; 35.7-80.2) |  | 9 | 9(100; 65.5-100) |  |
| Litter change interval | <10 days | 80 | 45(56.2; 45.3-66.6) | 0.664 | 45 | 40(88.9; 76.05-95.6) | 0.672 |
|  | 10-20 days | 30 | 16(53.3; 36.1-69.8) |  | 16 | 15(93.7; 69.7-100) |  |
|  | >20 days | 40 | 19(47.5; 32.9-62.5) |  | 19 | 16(84.2; 61.6-95.3) |  |
| Litter reuse | Yes | 135 | 73(54.1; 45.7-62.2) | 0.585 | 73 | 64(87.7; 78-93.6) | 0.324 |
|  | No | 15 | 7(46.7; 24.8-70) |  | 7 | 7(100; 59.6-100) |  |
| Litter dumping site | Near | 80 | 41(51.2; 40.5-62) | 0.585 | 41 | 35(85.3; 71.1-93.5) | 0.326 |
|  | Remote | 70 | 39(55.7; 44.1-66.7) |  | 39 | 36(92.3; 79-98.06) |  |
| Litter dumping type | Open | 90 | 48(53.3; 43.1-63.3) | 0.490 | 48 | 43(89.6; 77.4-95.9) | 0.899 |
|  | Pit | 45 | 26(57.8; 43.3-71) |  | 26 | 23(88.5; 70.2-96.8) |  |
|  | Burning | 15 | 6(40; 19.7-64.3) |  | 6 | 5(83.3; 41.8-98.9) |  |
| Sample source | Dumped litter | 30 | 16(53.3; 36.1-69.8) | 0.048 | 16 | 16(100; 77.3-100) | 0.007 |
|  | Feed | 29 | 15(51.7; 34.4-68.6) |  | 15 | 12(80; 54.05-93.7) |  |
|  | Litter | 30 | 12(40; 24.6-57.7) |  | 12 | 12(100; 71.8-100) |  |
|  | Soil | 31 | 14(45.2; 29.1-62.2) |  | 14 | 9(64.2; 38.6-83.8) |  |
|  | Water | 30 | 23(76.7; 58.8-88.5) |  | 23 | 22(95.6; 77.3-100) |  |
