# Supplementary material for: High prevalence of ciprofloxacin resistance in Escherichia coli isolated from chickens, humans and the environment: An emerging one health issue
Source: PLoS One. 2023 Nov 20;18(11):e0294043. doi: 10.1371/journal.pone.0294043 (PMC10659180; doi:10.1371/journal.pone.0294043)
Supplement: S3 Table — (DOCX) [file pone.0294043.s003.docx]

**S3 Table. Demographic data and prevalence of *E. coli* and ciprofloxacin resistant *E. coli* in hospitalized humans**

| **Explanatory variable** | **Covariable** | ***E. coli*** | | | **Ciprofloxacin resistant *E. coli*** | | |
| --- | --- | --- | --- | --- | --- | --- | --- |
|  |  | **N** | **Prevalence (%; 95% CI)** | **P-value** | **N** | **Prevalence (%; 95% CI)** | **P-value** |
| Age | < 18 years | 43 | 10(23.3; 13-37.9) | <0.001 | 10 | 9(90; 57.4-100) | 0.223 |
|  | 18-55 years | 55 | 26(47.3; 34.7-60.2) |  | 26 | 21(80.8; 61.7-91.9) |  |
|  | >55 years | 52 | 37(71.1; 57.6-81.7) |  | 37 | 35(94.6; 81.4-99.4) |  |
| Sex | Female | 61 | 25(41; 29.5-53.5) | 0.119 | 25 | 24(96; 78.9-100) | 0.17 |
|  | Male | 89 | 48(53.9; 43.6-63.9) |  | 48 | 41(85.4; 72.5-93.07) |  |
| Diarrhoea | Yes | 71 | 33(46.5; 35.4-58) | 0.611 | 33 | 30(90.9; 75.7-97.6) | 0.643 |
|  | No | 79 | 40(50.6; 39.8-61.4) |  | 40 | 35(87.5; 73.4-95.01) |  |
| Ciprofloxacin use | Yes | 107 | 59(55.1; 45.7-64.2) | 0.012 | 59 | 52(88.1; 77.2-94.4) | 0.611 |
|  | No | 43 | 14(32.6; 20.4-47.5) |  | 14 | 13(92.9; 66.5-100) |  |
| Drinking water source | Tube well | 75 | 36(48; 37.07-59.1) | 0.87 | 36 | 34(94.4; 80.9-99.4) | 0.145 |
|  | WASA | 75 | 37(49.3; 38.3-60.4) |  | 37 | 31(83.8; 68.5-92.7) |  |
