# Supplementary material for: High prevalence of ciprofloxacin resistance in Escherichia coli isolated from chickens, humans and the environment: An emerging one health issue
Source: PLoS One. 2023 Nov 20;18(11):e0294043. doi: 10.1371/journal.pone.0294043 (PMC10659180; doi:10.1371/journal.pone.0294043)
Supplement: S2 File — (PDF) [file pone.0294043.s006.pdf]

DISCOVER QGIS (../../ABOUT/INDEX.HTML)
FOR USERS (../../FORUSERS/INDEX.HTML)
GET INVOLVED / DEVELOPMENT (../INDEX.HTML)

- Write Documentation (../document.html)
- Translation (../translate.html)
- Development (../development/index.html)
- Tests (../tests.html)
- Daily Reports (../daily_reports/index.html)
- Plugins website (https://plugins.qgis.org)
- Mailing lists (../mailinglists.html)
- Project Organisation (../governance/index.html)
- Meetings (../meetings/index.html)
- Sustaining Membership Program (../governance/sustaining_members/index.html)
- Donations (https://donate.qgis.org)
- Certification (../certification.html)
- *FAQ*
  - *User*
    - *How to ask a QGIS question?*
    - *How are QGIS release names selected?*
    - *How to cite QGIS?*
    - *I created a map with QGIS, do I have to*

FAQ

Here we collect answers to question which come up very often.

We will start of with this one page, maybe restructure it into sections, or even more pages if needed

User

How to ask a QGIS question?

If you are going to ask QGIS related question via the mailing lists (../mailinglists.html#qgis-mailinglists), please provide enough information to help others easily understand what your problem is. Without a clear and precise question, it is very hard to answer for anybody or takes just too much time because the answer will be a question to you etc etc. You may provide information such as:

- preferably a descriptive title for your email
- which QGIS version you used at that moment (exact)
- what are you expecting to get
- if applicable, what have you tried and the result you got
- if your question is about something broken, you may also provide:
  - what exactly is the error (if possible: screendump, stacktrace, copy of error text)
  - which Operating System and version
  - how you installed it (osgeo4w? standalone-installer? own build?)
  - at what time did it break (reinstall, new install, system update, project changes)

Keep in mind that the more precise is your question, the quicker and more accurate can be the answer.

Note

In case of a broken function, you may give a look at QGIS issue tracker (https://github.com/qgis/QGIS/issues) before mailing to the list. More information at Bugs, Features and Issues (../development/bugreporting.html#qgis-bugreporting).

How are QGIS release names selected?

After a successful developer meeting in Zurich (Switzerland), we decided that the next release should be related to it. Since then, all releases have been named after locations of our developer meetings.

How to cite QGIS?

To cite QGIS in your piece of work, or for an assignment, please use citation type that can be more helpful:

Cite the QGIS project in general

QGIS.org, %Y. QGIS Geographic Information System. QGIS Association. http://www.qgis.org (http://www.qgis.org)

Cite the QGIS Developers Manual

QGIS.org, %Y. QGIS 3.28. Geographic Information System Developers Manual. QGIS Association. Electronic document: https://docs.qgis.org/3.28/en/docs/developers_guide/index.html (https://docs.qgis.org/3.28/en/docs/developers_guide/index.html)

Cite the QGIS Installation Guide

QGIS.org, %Y. QGIS 3.28. Geographic Information System Installation Guide. QGIS Association. Electronic document: https://github.com/qgis/QGIS/blob/master/INSTALL.md (https://github.com/qgis/QGIS/blob/master/INSTALL.md)

Cite the QGIS User Guide

QGIS.org, %Y. QGIS 3.28. Geographic Information System User Guide. QGIS Association. Electronic document: https://docs.qgis.org/3.28/en/docs/user_manual/index.html (https://docs.qgis.org/3.28/en/docs/user_manual/index.html)

Cite the QGIS Server Documentation

mention QGIS?

Can I open ECW files with QGIS?

Development

Can I compile QGIS myself?

Visual Style Guide (../styleguide.html)

DOCUMENTATION (../../../DOCS/INDEX.HTML)

QGIS.org, %Y. QGIS 3.28. Geographic Information System API Documentation. QGIS Association. Electronic document: https://docs.qgis.org/3.28/en/docs/server_manual/index.html (https://docs.qgis.org/3.28/en/docs/server_manual/index.html)

Cite the QGIS API Documentation

QGIS.org, %Y. QGIS 3.28. Geographic Information System API Documentation. QGIS Association. Electronic document: https://qgis.org/pyqgis/3.28/index.html (https://qgis.org/pyqgis/3.28/index.html)

Preferred format: BibTeX (https://en.wikipedia.org/wiki/BibTeX)

Example BibTeX entry:

```
<pre>
    @Manual{QGIS_software,
      title = {QGIS Geographic Information System},
      author = {{QGIS Development Team}},
      organization = {QGIS Association},
      year = {%Y},
      url = {https://www.qgis.org (https://www.qgis.or
g)  },
    }
</pre>
```

I created a map with QGIS, do I have to mention QGIS?

There is no requirement to mention QGIS for maps produced with it. It is of course really welcome if would like to add a note saying that the map was produced with QGIS. “Made with QGIS” or “Map created using the Free and Open Source QGIS” are good examples of such a note.

> **Note**
>
> Do not say ©QGIS as QGIS does not hold copyrights on your work.

Can I open ECW files with QGIS?

Yes you can… BUT depending on your Operating System, it is more or less difficult.

If you are under Windows and are using the OSGeo4w installer, it is included.

If you are using macOS, you can find the ECW plugin and install instructions at https://www.kyngchaos.com/software/frameworks (https://www.kyngchaos.com/software/frameworks)

For other instructions, eg look here: https://www.faunalia.eu/en/blog/2019-05-21_ecw_support (https://www.faunalia.eu/en/blog/2019-05-21_ecw_support)

Development

Can I compile QGIS myself?

Yes, compiling QGIS from source is possible whatever OS you use (Windows, OS X, Linux). Please read Building QGIS from source (https://github.com/qgis/QGIS/blob/master/INSTALL.md)

If not stated otherwise, all content is licensed under Creative Commons Attribution-ShareAlike 3.0 licence (CC BY-SA) (http://creativecommons.org/licenses/by-sa/3.0/)

Select graphics from The Noun Project collection (https://thenounproject.com)
